# Myalgia in 30 Patients with Suspected Myopathy

**DOI:** 10.3390/ijerph17072502

**Published:** 2020-04-06

**Authors:** Diana Lehmann Urban, Elizabeth Lehmann, Leila Motlagh Scholle, Torsten Kraya

**Affiliations:** 1 Department of Neurology, Ulm University, 89081 Ulm, Germany; diana.lehmann@rku.de; 2 Department of Neurology, University of Halle/Wittenberg, 06120 Halle/S., Germany; elizabeth.lehmann@gmx.de (E.L.); torsten.kraya@uk-halle.de (T.K.)

**Keywords:** neuromuscular disorder, pain threshold, myalgia, muscular pain-fasciculation syndrome, myopathy

## Abstract

Background: In patients with neuromuscular disorder, only little data of myalgia frequency and characterization exists. To date, only a weak correlation between pain intensity and pressure pain threshold has been found, and it remains enigmatic whether high pain intensity levels are equivalent to high pain sensitivity levels in neuromuscular disorders. Methods: 30 sequential patients with suspected neuromuscular disorder and myalgia were analyzed with regard to myalgia characteristics and clinical findings, including symptoms of depression and anxiety and pain- threshold. Results: A neuromuscular disorder was diagnosed in 14/30 patients. Muscular pain fasciculation syndrome (MPFS) without evidence for myopathy or myositis was diagnosed in 10/30 patients and 6/30 patients were diagnosed with pure myalgia without evidence for a neuromuscular disorder (e.g., myopathy, myositis, MPFS, polymyalgia rheumatica). Highest median pain scores were found in patients with pure myalgia and polymyalgia rheumatica. Pressure pain threshold measurement showed a significant difference between patients and controls in the biceps brachii muscle. Conclusion: Only a weak correlation between pain intensity and pressure pain threshold has been suggested, which is concordant with our results. The hypothesis that high pain intensity levels are equivalent to high pain sensitivity levels was not demonstrated.

## 1. Introduction

The International Organization for the Study of Pain (IASP) defines pain as “an unpleasant sensory and emotional experience associated with actual or potential tissue damage, or described in terms of an injury. In 2013, the European Neuromuscular Centre (ENMC) care workshop noted that pain and fatigue are common symptoms in neuromuscular disorders (NMD) with a prevalence of 30–90%, present in all types of NMD, both in adults and children [1]. Jensen et al. stated that pain has a strong impact on different activities of daily life, e.g., mobility, work, school, leisure, and sleep [2]. Different data regarding the characterization of myalgia in patients with defined muscle diseases exist. From clinical observation, it is already known that myalgia occurs in different muscle disorders, e.g., proximal myotonic myopathy (PROMM) [3], occulopharyngeal muscular dystrophy (OPMD) [4], myositis [5], statin-induced myopathy [6], and mitochondrial myopathy [7]. In many of these, myalgia is well known as one of the main symptoms. However, only a small amount of data describing the frequency and characterization of myalgia in patients with neuromuscular disorders exists. The aim of this study was the clinical characterization and measurement of myalgia in patients with suspected neuromuscular disorder and the association to depression and anxiety symptoms. Furthermore, the pain threshold was quantified with a pressure algometer. To date, only a weak correlation between pain intensity and pressure pain threshold has been found, and it remains enigmatic as to whether high pain intensity levels are equivalent to high pain sensitivity levels in neuromuscular disorders. However, it is possible that the cause of myalgia is related to lowered pain thresholds.

## 2. Experimental Section

### 2.1. Patients and Controls

Thirty patients (25 female, 5 male; 21–69 years, mean: 50.5 years) with suspected neuromuscular disease and myalgia were included in the study (Table 1). All patients were included through the neurological outpatient department, the specialized muscular center and the neurological general ward of the Department of Neurology from the medical University Halle-Saale (Germany), as part of an already-planned outpatient follow-up or inpatient treatment during the years 2015–2016. The patients were referred to the clinic for further diagnostics by a specialist in neurology. Inclusion criteria were suspected neuromuscular disorder (either newly developed or progressive paresis, muscular atrophy, elevated creatine kinase (CK)-levels or in some cases myopathic changes in the electromyographic (EMG)) and myalgia. As controls served 26 individuals (12 female, 14 male; 31–70 years, mean: 53 years) without myalgia and in whom a neuromuscular disease has been excluded by clinical and electrophysiological findings. The ethical statement was approved during study design from the ethical committee from the University of Halle-Wittenberg. 

### 2.2. Questionnaires and HADS

In addition to demographic information (age and gender), information on myalgia localization and pain character, adjusted according to the McGill questionnaire (e.g., dull, cramping, stinging, burning, tension-like, drawing, raging, muscle ache-like, oppressive) [8] were collected from each patient. Furthermore, data according age of onset, progress of myalgia, provocation factors and whether cramps and/or fasciculation were observed during pain, so as CK elevations (norm values for CK (creatine-kinase): female < 2.85 μkat/L, male < 3.20 μkat/L), EMG findings and results of muscle biopsies were collected. The usual tender points [9] were scanned and documented. All patients ranked their pain sensation according to the visual analogue scale (VAS) [10] and completed the Hospital Anxiety Depression scale (HADS) to assess for depression (HAD-D) and anxiety (HAD-A) [11].

### 2.3. Pressure Pain Threshold

Pressure pain detection threshold (PPDT) (10 kg × 100 g) was determined by algesiometry in patients and controls. Measurements were performed using a Wagner Pain Test™ Model FPK Algometer (Wagner Instruments, Greenwich, CT, USA) over the muscle belly of the biceps brachii muscle and the quadriceps muscle (rectus femoris muscle), both on the right and left side. The measurement was repeated three times over each muscle, and means were calculated for each muscle and patient. 

### 2.4. Muscle Histopathology and Molecular Genetic Studies

Histopathological analysis of muscle biopsies from either the biceps brachii muscle or the vastus lateral muscle were performed according to standard protocols [12]. Genetic studies were performed according to standard protocols [13,14,15].

### 2.5. Statistical Analysis

Statistical analysis, calculation and visualization were performed using Prism 7 (GraphPad, San Diego, CA, USA). The analysis of a possible relationship between two variables was carried out using linear regression. Analysis of correlation was carried out using multiple, unpaired t-tests. The p-value selection was based on the FDR (False Discovery Rate). Significance was set to *p* = 0.05. The statistical tests chosen were predetermined by the size of the study group and the numerical range of values.

### 2.6. Standard Protocol Approvals and Participant Consent

Data acquisition and analysis was performed in compliance with protocols approved by the Ethical Committee of the Martin Luther University Halle-Wittenberg (ethical approval number 2015-18). Written informed consent was obtained from all participants prior to study inclusion.

## 3. Results

### 3.1. Muscle Diseases

Thirty patients with suspected neuromuscular disorder and myalgia were analyzed. The majority of patients (n = 14; female = 11, male = 3; median age 60 years) were diagnosed with a neuromuscular disorder: (I) four patients with clarified diagnosis: limb girdle muscular dystrophy type 1 B (LGMD1B) (n = 1), myoadenylate deaminase deficiency (MADD) (n = 1), anoctamin5- myopathy (n = 1) and mitochondrial myopathy without a genetic cause (n = 1); (II) six patients with myopthy (only evident by means of biopsy without nosological diagnosis); (III) two patients with polymyositis and (IV) two patients with polymyalgia rheumatica (PMR). In this group of patients with neuromuscular disorders (n = 14), EMG showed myopathic changes in 5/14 patients, elevated CK was observed in 4/14 patients and myopathic changes in muscle biopsy was found in 12/14 patients. 

Ten patients were diagnosed with muscular pain fasciculation syndrome (MPFS) (female = 7, male = 3; median age 47.5 years). Neither the EMG nor the muscle biopsy showed (performed in 7/10 patients) myopathic changes. CK elevation was shown in two patients. 

The third group consists of patients (n = 6; female = 6; median age 46 years) with pure myalgia without any evidence for neuromuscular disorders. EMG, CK and muscle biopsy (performed in 4/6 patients) showed normal results.

### 3.2. Localization of Myalgia

The majority of patients (30.43%) analyzed in this study described myalgia in two locations (lower and/or upper extremity, face, neck, trunk). The highest prevalence was shown in the lower and upper extremities. Unilateral pain was not reported. Interestingly, all patients described myalgia in the lower extremities. Furthermore, only one patient mentioned to have myalgia located in the facial area.

### 3.3. Description of Myalgia

Cramping pain was described by the majority of patients (40%), followed by burning (33%), stinging and dull (27%), drawing (16%), muscle ache-like (10%) and tension-like (10%) pain. One patient reported oppressive pain. 93% of patients described pain during physical activity; however, 73% of patients described muscle pain during resting without prior activity. Only 17% of patients reported that myalgia could not be triggered.

### 3.4. Pain Scores

The highest median VAS-score (8/10) was reported in the myalgia-group (n = 6), followed by VAS 7/10 in patients with muscular pain fasciculation syndrome (n = 10). The lowest median VAS-score (6/10) was found in patients with neuromuscular disorders (n = 14). There was no significant difference between the groups (p = 0.57).

### 3.5. Pressure Pain Threshold

**Figure 1:**
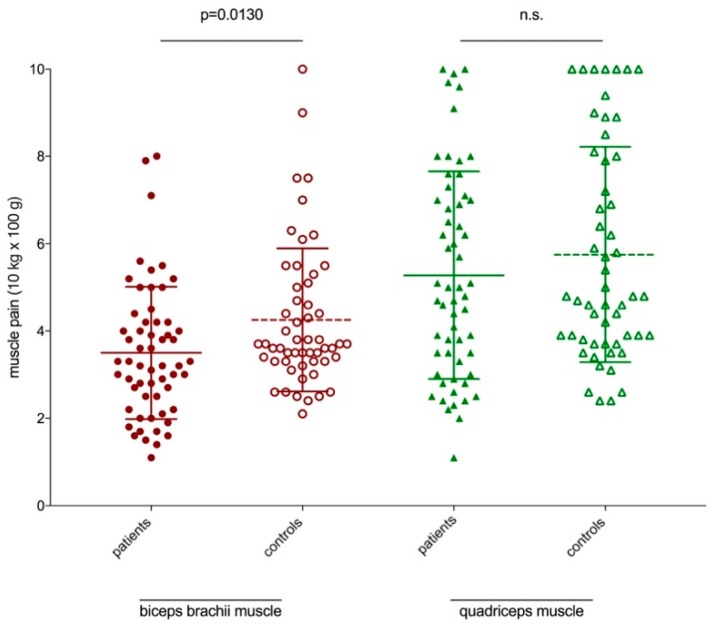
Pressure pain detection threshold measurement of patients (filled forms) and controls (open difference between patients and controls (p = 0.0130) in the biceps brachii muscle. Graphs show mean with standard deviation (SD), analysis using unpaired t-test.

**Figure 2:**
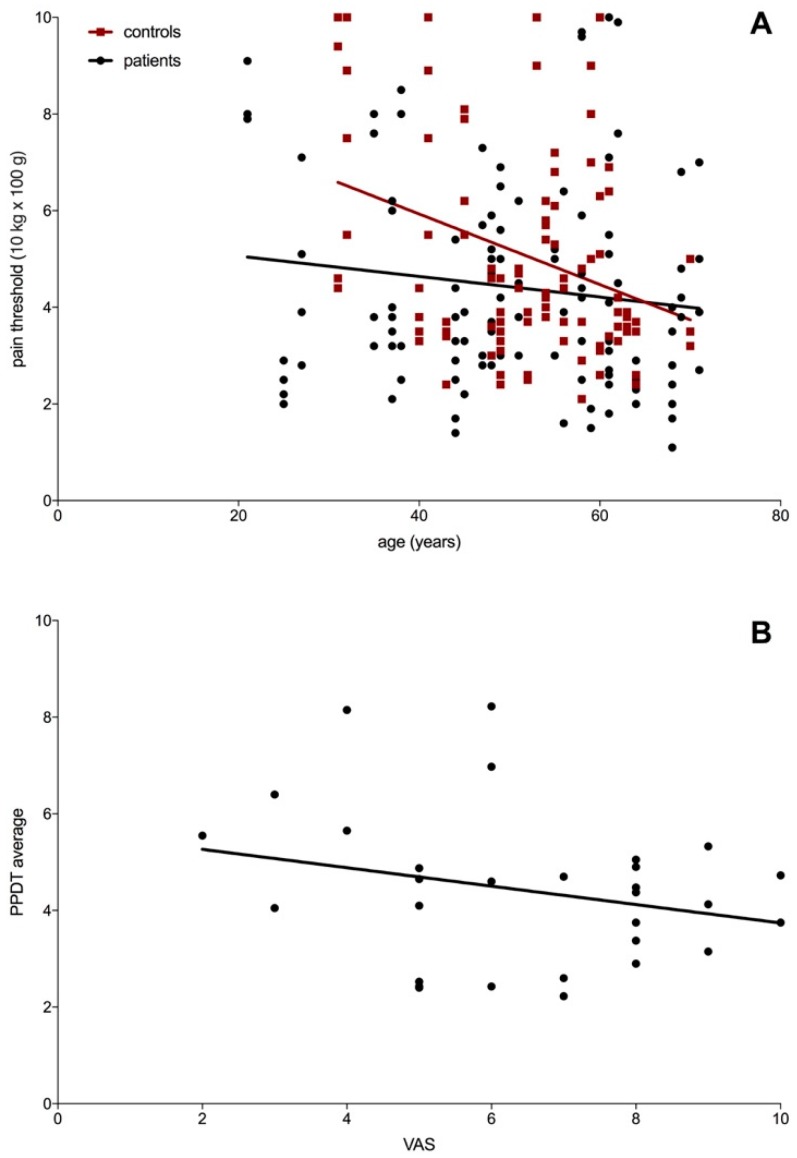
(**A**) Correlation of pressure pain detection threshold (PPDT) with the age of patients (circle) and controls (square). The PPDT decreases in both, patients (n.s., r^2^ = 0.1) and controls (p = 0.0010, r^2^ = 0.017) with age. (**B**) Correlation of VAS ranking and the PPDT average (n.s., r^2^ = 0.065), analysis using linear regression.

PPDT measurement showed a significant difference between patients and controls (p = 0.0130) in the biceps brachii muscle. However, the PPDT for the quadriceps muscle revealed no significant differences in patients and controls (Figure 1). The PPDT was correlated with the age of patients and controls while pain threshold measurement (Figure 2A). The PPDT decreases in both patients (n.s.) and controls (p = 0.0010) with age. However, there was no correlation of the patients VAS ranking and the PPDT average (n.s.) (Figure 2B). PPDT was not significantly (p = 0.0819) different in the upper and lower extremities in patients with myalgia in these regions. There was no side difference (p = 0.9769) in PPDT measurement. 

### 3.6. HADS

The majority of patients showed normal results in HADS evaluation (HADS-A: 20 patients; HADS-D: 19 patients) (Table 1); however, there were several patients with suspect or conspicuous test results. 4 of 30 patients showed HADS-Anxiety Score >10 with median VAS pain score of 6.4/10 and median age of 51 years. 3/30 patients showed HADS-Depression scores >10 with median VAS pain score of 9/10 and median age of 44 years. 2/30 patients showed HADS-anxiety and HADS-depression score >10 with median pain score VAS 9/10 and median age of 47.5 years. 

In the group of patients with a neuromuscular disorder, the median scores were 4 for anxiety and 6 for depression, in patients with muscular pain fasciculation syndrome 4.5 and 3.5 and in the myalgia group 8.5 and 5. There was no significant difference between the patients with higher scores for anxiety and depression in the different groups and between the pain scores.

## 4. Discussion

Based on several studies, it is already known that myalgia is a common feature in neuromuscular diseases [1]; however, only a small amount of data describing pain localization and characterization exists. In a group of 30 patients with suspected neuromuscular disease and myalgia, most of the patients were diagnosed with a neuromuscular disorder and less frequently with muscular pain fasciculation syndrome or pure myalgia. In the largest group of patients (neuromuscular disorder, n = 14), a clarified diagnosis was established in four patients. In 43% of patients, the diagnosis was based on myopathic changes in EMG and muscle biopsy; however, without nosological assignment. The group of muscular pain fasciculation syndrome- patients (33% of all patients) showed an CK elevation in 14%. Normal results were seen in both, EMG and muscle biopsy. The smallest group consisted of patients with pure myalgia (n = 6) without any evidence for a muscular disease.

All patients described myalgia in the lower extremities, 43% in two localizations. However, the highest prevalence was shown in the lower and upper extremities. Patients reported myalgia as cramping, burning and stinging/ dull (40%, 33%, and 27% respectively). In 93%, myalgia was provoked by physical activity; however, in 73% during rest. However, the highest pain scores were reported in patients with pure myalgia and polymyalgia rheumatica and notes for depression and anxiety. In contrast, there were significant differences in pain threshold only in the biceps brachii muscle. 

Thirty-four patients with genetically confirmed myotonic dystrophy type 2 (DM2), 28 patients with fibromyalgia (FMS), and 33 healthy controls were included into an explorative study, assessing qualitative and quantitative aspects of pain in DM2 [16]. Pain prevalence was 65% in DM2, 100% in FMS (*p* < 0 .001), and 15% in healthy controls (*p* < 0.001). Consistent with the results of our study, the mean pressure pain thresholds were lower in DM2 patients than in healthy controls; however, no differences were found in electric pain thresholds between DM2 and healthy controls [16]. According to the 184th ENMC International Workshop for pain and fatigue in neuromuscular disorders, pain scores were independent of age, impairments, physical activity level, or muscle force [1]. A recent meta-analysis [17] revealed that, indicated by a large effect size, the pain threshold increases with age in healthy adults, which is in contrast to our study results. However, in the present study, patients harboring a neuromuscular disease were investigated. Petrini et al. [18] showed that the PPDT significantly decreased with age, which is in agreement with our findings. It has been suggested that pain experiences in the elderly differ from the experiences in the young and that the elderly may also appraise pain experiences using different psychological strategies [18]. The results of our study indicate that these suggested coping strategies were applied by elderly diseased people, too. The fact that the PPDT measurement of the quadriceps muscle revealed no significant differences in patients and controls could be due to the larger muscle size in comparison to the smaller biceps brachii muscle.

In a recent study, only a weak correlation between pain intensity and pressure pain threshold was found [19]. This is interestingly concordant with our results, showing no correlation of the patients VAS ranking and the PPDT average (Figure 2B). Another group came to similar results: patients with temporomandibular disorders were investigated to correlate visual analogue scale (VAS) and pressure pain threshold (PPT) [20]. The hypothesis that high pain intensity levels are equivalent to high pain sensitivity levels was not demonstrated [20], according to our study results. It has already been suggested that other factors are clearly important in explaining pain experience, including the contribution of central nervous system nociceptive processes and psychological variables to the maintenance of chronic pain [19]. However, the highest median VAS score was found in patients with pure myalgia and polymyalgia rheumatica. In contrast, patients with diagnosis of pure myopathy and polymyositis showed the lowest median VAS- scores. 

In 1/3 of the investigated patients, we found no pathological findings in neurological examination, histopathological investigation and additional diagnostics. According to their clinical manifestation of symptoms, muscular pain-fasciculation syndrome was diagnosed, suggesting that this disease should be considered as differential diagnosis more often. 

## 5. Conclusions

Recent studies have suggested only a weak correlation between pain intensity and pressure pain threshold, which is concordant with our results. The hypothesis that high pain intensity levels are equivalent to high pain sensitivity levels was not demonstrated. Furthermore, we did not find pathological evidence in neurological examination, histopathological investigation or additional diagnostics in 1/3 of the investigated patients. According to their clinical manifestation of symptoms, muscular pain-fasciculation syndrome was diagnosed, suggesting that this disease should be considered more often as a differential diagnosis. 

We are aware that this manuscript has its limitations, especially due to the small sample size. However, in times of increased genetic testing, e.g., next-generation sequencing (NGS), it is still difficult to find patients with myopathies, as they are still regarded as “rare diseases”. Furthermore, the study group was limited due to the fact that we performed electrophysiological studies such as muscle biopsy in nearly all patients (23/30). Upcoming studies need to investigate a larger patient cohort. This might provide more information about myalgia in patients with defined neuromuscular disorders. 

## Figures and Tables

**Table 1:** Demographic and clinical information, HADS results, and VAS-ranking such as diagnosis of patients included in the study, norm values for CK (creatine-kinase): female < 2.85 μkat/L, male < 3.20 μkat/L. n.p. = not performed, PMR = polymyalgia rheumatica, MPFS = muscular pain fasciculation syndrome.

Patient-ID	Age at Study Inclusion	Gender (m/f)	HADS-A/D		VAS (x/10)	EMG	CK (μkat/L)	Biopsy	Diagnosis
1	61	f	4	8	8	myopathic	8.42 **↑**	myopathic	muscle dystrophy (LGMD 1B)—heterozygote mutation in the *LMNA*-gene (c. 1930C<T)
2	25	f	5	1	5	myopathic	42.45 **↑**	myopathic	anoctaminopathy–homozygote mutation in the *ANO5*-Gen (c.191dupA)
3	59	m	3	1	6	normal	<3.20	myopathic	myoadenylate deaminase deficiency
4	71	f	2	4	5	normal	<2.85	myopathic	mitochondrial myopathy
5	35	f	7	6	4	myopathic	6.81 **↑**	myopathic and neurogenic	myopathy
6	58	f	8	9	6	normal	<2.85	myopathic	myopathy
7	64	m	3	1	6	normal	<3.20	myopathic	myopathy
8	68	f	4	4	5	normal	<2.85	myopathic	myopathy
9	45	f	12	8	8	normal	<2.85	myopathic	myopathy
10	37	f	4	15	9	normal	<2.85	myopathic	myopathy
11	68	f	3	3	7	normal	<2.85	n. p.	PMR
12	61	m	9	10	5	normal	<3.20	n. p.	PMR
13	51	f	15	17	8	myopathic	<2.85	myopathic/inflammatoric	polymyositis
14	62	f	3	9	3	myopathic/myotonic	5.2 **↑**	myopathic/inflammatoric	polymyositis
15	61	m	1	1	4	normal	<3.20	normal	MPFS
16	44	f	3	0	7	normal	<2.85	normal	MPFS
17	49	f	7	4	5	normal	<2.85	normal	MPFS
18	69	f	8	4	8	normal	<2.85	n. p.	MPFS
19	27	f	7	5	10	normal	<2.85	normal	MPFS
20	21	m	7	5	6	normal	<3.20	normal	MPFS
21	49	f	4	3	9	normal	7.82 **↑**	normal	MPFS
22	47	m	5	5	7	normal	<3.20	n. p.	MPFS
23	38	f	1	0	2	normal	4.19 **↑**	normal	MPFS
24	48	f	1	1	8	normal	<2.85	n. p.	MPFS
25	56	f	2	3	8	normal	<2.85	normal	myalgia
26	44	f	17	20	10	normal	<2.85	normal	myalgia
27	48	f	9	5	9	normal	<2.85	normal	myalgia
28	37	f	6	5	8	normal	<2.85	n. p.	myalgia
29	58	f	9	8	5	normal	<2.85	normal	myalgia
30	55	f	8	5	3	normal	<2.85	n. p.	myalgia
